# “No One Really Likes Crying in School”: The Influences of Classroom and Institutional Dynamics Upon Student Absenteeism During COVID-19

**DOI:** 10.5334/cie.43

**Published:** 2022-08-25

**Authors:** Andrew Louis Kipp

**Affiliations:** 1 Texas A&M Higher Education Center at McAllen, US

**Keywords:** Student absenteeism, ecological agency, COVID-19, school improvement, institutional practices, classroom practices

## Abstract

The COVID-19 pandemic has worsened the already problematic issue of student absenteeism. This study uniquely employs an ecological agency approach to better understand student absenteeism during COVID-19. Using a case study methodology, the study captures the experiences of two absentee students within a United States suburban high school during the 2020–2021 school year to better understand the institutional structures motivating their daily decision to attend or miss school. In the remote learning environment, rigorous curricular expectations, minimal social interactions, teacher-led instruction as a response to student disengagement from student-led instruction, and lower teacher expectations contributed to the participants’ daily decision to miss school. In the remote, hybrid, and full-time in-person learning spaces, staff apathy toward bullying, minimal space to escape feelings of anxiety, and fewer tutoring outlets motivated student absenteeism. Therefore, the school environment can better promote attendance during COVID-19 by establishing an in-school space to escape heightened anxiety, academic supports to reduce grade-induced anxiety, shifting from nonintervention to prosocial instructional interventions in all learning environments, teacher voices in policy design, reducing teacher-led instruction, and shifting teacher beliefs to an asset mindset. Recommendations for future research are included.

Student absenteeism, commonly defined as missing 10% or more of school days during the school year (Balfanz & Byrnes, 2012), is a global issue in education. Before the start of the COVID-19 pandemic, 17.8% of students globally engaged in absenteeism, with secondary-age students, low socioeconomic students, female students, urban students, and ethnic minority students being more likely to engage in absenteeism (Birioukov, 2015; Gottfried, 2015; Kearney et al., 2019). Student absenteeism leads to lower short-term academic, emotional, and social growth (Gottfried, 2010, Gottfried, 2014) and also presents long-term problematic outcomes including criminal activity, lifelong legal issues, unemployment, dropping out of school, drug usage, violence, depression, and criminalization (Attendance Works, 2017; Attwood & Croll, 2006; Garry, 1996; Hibbett & Fogelman, 1990; Mallett, 2016). In addition, student absenteeism is harmful to schools because attendance records are often used as a measurement of academic success and achievement; further, high absenteeism rates lead to significant financial burdens for schools (Gottfried & Hutt, 2019; Hutt, 2018).

Research in student absenteeism and similar fields (e.g., truancy, permanent student absenteeism/dropping out, school refusal) has endeavored to improve absentee behaviors that are within students’ control and will not correct themselves without assistance (Heyne et al., 2019). When students engage in absenteeism, schools either punish them, provide interventions based on students’ unique needs, or ignore the behavior completely. With apathy and punitive action being ineffective at resolving student absenteeism, and often dangerous toward minority students due to their overrepresentation with regard to receiving punitive actions, research has focused on targeting student needs and providing interventions to change student behavior (Amemiya et al., 2020; American Psychological Association Zero Tolerance Task Force, 2008; Kearney, 2008).

Globally, over 850 million children were in a remote learning setting as a response to the COVID-19 pandemic (UNESCO, 2020). This led to disruptions with instruction, curriculum, practices, and policies (Daniel, 2020); it also resulted in decreasing student attendance rates (Coker, 2020; Nathwani et al., 2021). However, there is minimal research examining the influences of institutional and classroom conditions upon student absenteeism during COVID-19, specifically upon the absentee student’s daily decision to attend or miss school.

Student absenteeism research utilizes a variety of approaches, perspectives, and frameworks to capture the specific needs of absentee students, and in turn, synthesize interventions based on these needs (Havik & Ingul, 2021). For example, the psychological perspective focuses on the mental health needs of students (Kearney, 2008). The criminal justice perspective targets the broader societal factors and rule-breaking behaviors, whereas the educational perspective focuses on self, parent, family, peer, school, and community factors (Kearney, 2008). The Kids and Teens at School (KiTeS) framework (Melvin et al., 2019) further elaborates on the self, parent, family, peer, school, and community factors and includes the microsystem (which interacts with the student) and the mesosystem (which is the interrelationship between two factors such as the parent and school); the goal of the framework is to improve the microsystem and mesosystem, which may improve attendance. The Response to Intervention model has also been incorporated as an approach to promote school attendance through individualizing interventions based on student needs (Kearney & Graczyk, 2014). Gentle-Genitty et al.’s (2020) framework utilizes attendance records and markers to proactively implement individually focused interventions before the absenteeism worsens. School bonding analyzes the number of connections between the student and school; interventions focus on improving the number of connections between the student and school (Keppens & Spruyt, 2020). Finally, a(n) voluntarily/involuntarily framework prioritizes on the different conditions that are within the absentee student’s control (Birioukov, 2015). Individually, each of the above frameworks offers a different perspective of the complex issue of student absenteeism and uncovers unique contextual conditions or a collection of conditions that lead to absentee behaviors.

Another theoretical framework, the ecological agency theoretical framework, magnifies the environment-actor interplay in relation to human decision-making (Biesta & Tedder, 2006, 2007) and provides a unique, but viable, avenue to understanding how the COVID-19 environmental conditions influence absentee behaviors, as further described in the following.

## Viewing Absenteeism Through the Ecological Agency Theoretical Framework

**Figure 1:**
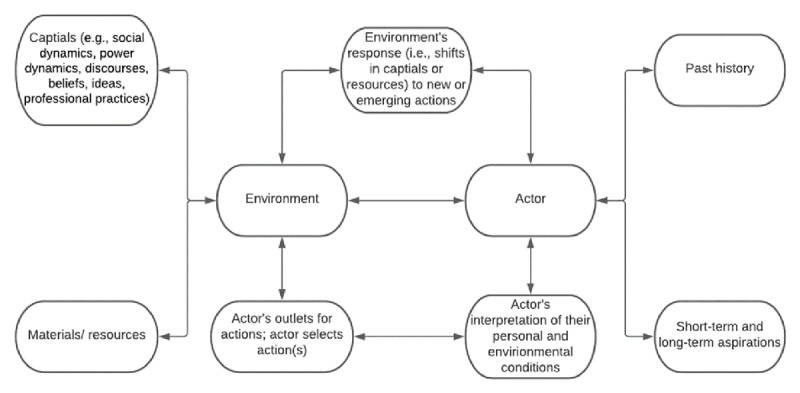
Actor-Environment Interplay. *Note*: Adapted from Biesta et al. (2015).

The ecological agency theoretical framework is a refined conceptualization of agency theory. Historically, agency theory focuses on human action and the unique conditions that motivate any particular action (Emirbayer & Mische, 1998). Biesta’s and Tedder’s (2006, 2007) concept of agency recognizes that agency is a concrete action *achieved* within an environment, which differentiates it from past iterations of agency by developing an interpretation of the actor-environment interplay. That is, the person reflects upon their outlets for actions based on their temporal dimensions – which include their past history (iterational dimension), future aspirations (projective dimension), and environmental capitals (e.g., social dynamics, power dynamics, discourses, beliefs, ideas, professional practices) and materials (practical-evaluative dimension) – and selects potential actions based on their interpretation and reflection upon their unique conditions (Biesta & Tedder, 2006, 2007; Biesta et al., 2015, 2017; Priestley et al., 2015). The person’s manifested action either follows or resists the unique environmental factors (Bridwell-Mitchell, 2015). In turn, the environment, which is interested in specific actions and outcomes, shifts the capitals and materials to afford/constrain certain behaviors thereby creating a feedback loop between the environment and actor (Biesta et al., 2015). Figure 1 summarizes the ever-changing interplay between the environment and the actor.

Juxtaposing the ecological agency framework with the context of the present study, I analyzed how the school’s dynamics (e.g., teachers’, administrations’, and policymakers’ actions, beliefs, policies, instruction, curriculum, and practices) influenced students’ daily decision to attend or miss school during the 2020–2021 school year for the purpose of helping the school environment better promote student attendance.

Educational research uses the ecological agency theoretical framework to understand how educational policy and curriculum influence teacher action (e.g., Biesta et al., 2015; Bridwell-Mitchell, 2015; Calvert, 2016; Jenkins, 2019; Morley, 2019; Robinson, 2012; Tao & Gao, 2017). While the use of the ecological agency framework to understand absentee student decision-making has been theorized (Kipp & Clark, 2021), to date no publications have implemented it in practice to inform the supports and capitals that influence absentee students’ daily decisions to attend or miss school. Therefore, the present study captured, through the ecological agency theoretical framework, the personal and environmental conditions of absentee students and how the environmental dynamics (e.g., collective or individual beliefs, practices, policy, instruction, and/or curriculum) and personal factors shift the absentee students’ daily decision to attend or miss school.

The research questions were as follows:

1. What were the influences that determined students’ absenteeism during the COVID-19 pandemic?
2. How did the institutional collective or individual beliefs, practices, policy, instruction, and/or curriculum, along with other personal experiences, influence absentee students’ daily decisions to attend or miss school during the COVID-19 pandemic?

## Setting and Participants

The study was conducted in a suburban school in the United States. The school provided students a remote learning environment from September 2020 to the first week of February 2021 as a response to the COVID-19 pandemic. In preparation for remote learning, teachers received three weeks of professional development about the best online practices prior to the start of the school year due to their lack of experience teaching online. Within the remote learning format, teachers and students engaged in a block schedule format (i.e., four classes per day; eight total classes per two days) with 83-minute synchronous class periods.

During the second and third weeks of February 2021, the school shifted to a hybrid learning model, in which half the students met in-person and the remaining students met in a remote asynchronous setting for four of their eight daily classes. The following day, the students switched from online to in-person and vice versa for the same four classes. On their remote day, the students potentially had asynchronous classwork to complete. Finally, the students repeated the same cycle over the next two days with their remaining four classes. Thus, they rotated through their classes over the course of four school days.

During the final week of February 2021, the school moved back to a full-time in-person block schedule and maintained this format for the rest of the school year. Within the in-person learning settings, new COVID-19 migration practices were added (e.g., social distancing, wearing masks properly, wiping down desks after use). Finally, all learning environments included a daily 30-minute seminar for the purpose of providing a space for students to work on homework or receive online tutoring via Zoom from the school’s teachers.

During remote learning, students were expected to log onto their online classes via Zoom, engage in the learning activities, and complete homework. In hybrid, the expectation was for students to participate with the in-person learning activities, adhere to the COVID-19 mitigation practices during in-person learning, engage in asynchronous learning activities if provided during their remote day, and complete homework. Finally, the expectation for full-time in-person learning was for students to participate with the learning activities, comply with COVID-19 mitigation practices, and complete any homework. Throughout, teachers were asked to provide instruction for their 83-minute classes and teach previous years’ mandated curriculum regardless of the setting.

To be included in the study, students had to be enrolled as a student within the study’s site, be missing 10% or more of school days, complete an assent form, and volunteer to share their experiences within each learning environment during the 2020–2021 school year. The study captures the personal and school experiences of two students who met the delimitations. All participants are anonymous, and pseudonyms are used. The two students’ pseudonyms are Marble and Tessa.

## Research Design

The study utilized a case study design (Merriam, 1998; Stake, 1995) to explore what personal and environmental factors influenced participants’ daily attendance decision, and how the environment shifted their daily attendance decisions. A case study design was selected because agency is captured when participants critically reflect upon their contemporary and past actions, experiences, and aspirations (i.e., the temporal dimensions); the data collection sources were synthesized to promote reflection upon the temporal dimensions (Biesta & Tedder, 2006; Biesta et al., 2017).

Participating students engaged in three data collection activities, consisting of interviews (Biesta & Tedder, 2006; Creswell & Poth, 2018), relational maps (Copeland & Agosto, 2012), and drawings (Guillemin, 2004; Literat, 2013). Further, prolonged observations at the site of study were used to ensure triangulation of data (Houghton et al., 2013). Specifically, prolonged observations were achieved through classroom and school walkthroughs; interviews with two of the participants’ peers, four teachers, three counselors, and two administrators; and analytical memos during the walkthroughs and interviews. These elements enriched an understanding of the environmental dynamics within each learning environments (Houghton et al., 2013).

Creswell’s and Poth’s (2018) procedure for analyzing qualitative data was used, which includes coding the data, condensing the codes into categories, conceptualizing the categories into themes, and, finally, connecting the themes to the overarching case and research questions.

First, the audio data from the interviews were transcribed, read through multiple times, and subjected to iterations of in vivo and inductive coding strategies to understand the data because participants’ *interpretation* of their temporal dimensions is an important feature of the environment-actor interplay (Biesta et al., 2015; Braun & Clarke, 2006; Creswell & Poth, 2018; Saldaña, 2021). In vivo and inductive coding features the person’s perspective, thereby providing a richer understanding of their actions compared to deductive coding, which presents prescribed codes from the researcher and not the participant (Linneberg & Korsgaard, 2019). The coding focused on understanding what factors led to students’ attendance decisions and how the environment shifted their decisions (Biesta & Tedder, 2007). Coding was completed by chunking to prevent code proliferation (Saldaña, 2021).

Second, the codes were collapsed into categories based on their similarities (Saldaña, 2021; Xu & Storr, 2012) and, finally, organized into themes to generate overarching themes that explain the research questions (Braun & Clarke, 2006; Creswell & Poth, 2018).

Other data sources and artifacts (i.e., staff and student interviews, analytical memos, concept maps, drawings) were used throughout the thematic analysis to support the emergence of codes, categories, and themes and to ensure triangulation, trustworthiness, and rigor (Creswell & Poth, 2018; Houghton et al., 2013). Additional strategies to promote rigor and trustworthiness during data collection and analysis included peer debriefing, member checking, audit trail, reflexivity, and thick descriptions (Houghton et al., 2013). The research was approved by the Institutional Review Board.

## Results

### Lessons Learned About the Factors Influencing Student Absenteeism During COVID-19

The following section represents the findings from the two participants during the data collection and analysis. Each theme illustrates the lessons learned about the factors that motivated their daily attendance decision. The findings also address how the specific environmental and personal conditions shifted students’ individual daily action toward (or against) student absenteeism or school attendance. To ensure rigor and triangulation, teachers’, administrators’, students’, and counselors’ quotes were added to reinforce the findings and illuminate the actor-environment interplay from the ecological agency framework.

### Tessa

#### Social Capital (Peer Interactions)

Both positive and negative peer interactions influenced Tessa’s daily attendance decision. She shared:

Sometimes motivating me to attend school was who’s going to be there and like which friends can I hang out with during school. So, like, it’s easier when you start to think about that. But it’s still difficult, like hearing that so-and-so talked behind your back about something you didn’t even do or who you are. It’s like up and down.

Tessa, a person of color, was bullied throughout her school career for being different in her predominantly white schools. As she got older, these experiences continued but shifted over to online bullying on mediums such as Snapchat. She expanded upon her bullying experiences:

I used to be called the N-word every day. And so, it got to the point that I really thought it was normal. [Now,] I have other friends, and they’ve been making fun of me because I’m too whitewashed … And I’m mixed. So, [I’m] not feeling Black enough for the Black community and not feeling white enough for the white community. It separates me to where like I feel like I’m just alone. So, what’s the point in trying?

It was hard because I felt different again from all the kids. And they always, like … They wouldn’t, like, come out and say it, but they always thought I was different. And like, I didn’t think I was as worthy as the other kids in the classes.

Tessa was distrustful of her peers because even her close friends teased her for being different. If there was a bully in her class, regardless of the learning environment (i.e., full-time in-person, hybrid, or remote), it made her uneasy. For example, in the online space, she did not have her video camera turned on. Even then, having her name on the screen felt threatening to her because she thought her bullies would find her name and message her. In the remote, hybrid, and full-time in-person learning environments, she shared that, “Like, that’s rude [when receiving disparaging remarks,] but I would never stick up for myself. So, at that point, like, I was more aware of the harm that they were putting in my brain. So, I wanted to stay home or log off.” She would not report her bullying experiences to staff members. One of the counselors shared their perspective on why students do not report bullying:

I feel like students don’t tell, so counselors don’t get involved. But if we do, we support [the bullied student] and add the administration [to help intervene.] From there, student conferences and fact finding [occur.] We ask questions like, ‘Is it possible that you misinterpreted the situation?’ [or] ‘Did it happen more than once?’ Then we have a conference with the bully. Then, we conference with the parents. Many times, things are misinterpreted. Nobody has power over the other, and there is no bullying; it’s like retaliation as a response [to criticism.] 90% of bullying is not bullying because both parties need to be accountable.

To that end, Tessa learned that the school did not believe her. She felt her bullying got ignored. It made her distrustful of the school and shifted the responsibility of bullying onto her. The bullying primarily harmed her self-worth, which manifested in negative self-talk. When she was in emotional pain from bullying, she looked for a space to improve her mental health. During remote learning, without nearby friends who could console her, she either logged off Zoom or lied to her teachers by telling them that her internet was problematic thus making attendance impossible. This enabled her to focus on anxiety-reducing activities.

Compounding the issue was the staff’s narrative of bullying during remote learning. One teacher shared that, “There was so little dialogue between students and teachers. I have no idea if bullying happened or not. I couldn’t see it at least.” One of the counselors remarked, “Looking back at remote learning, I feel like there were fewer instances of crises just because we weren’t there logistically.” Tessa’s bullying went unnoticed during remote learning.

Starting during hybrid learning, Tessa was re-motivated to attend because her friends were there. However, so were her bullies. She knew that going to the counselor’s office would put in her a space where no bullies would be. However, counselors were rarely available, and if they were, she simply used the space to escape her bullies. A counselor expanded upon their availability by sharing, “What I saw when we went back [to hybrid learning] was a large number of students showing up due to mental health [crises.]” So, if no counselors were available, Tessa retreated to the bathroom and cried, which embarrassed her and devastated her self-worth. On the most painful days, rather than crying in the bathroom, she used the side doors of the school to leave because she felt nobody would notice her. As Tessa put it, “No one really likes crying in school.” She was never caught from leaving school this way.

#### Material Resources (Availability of Help)

**Figure 2:**
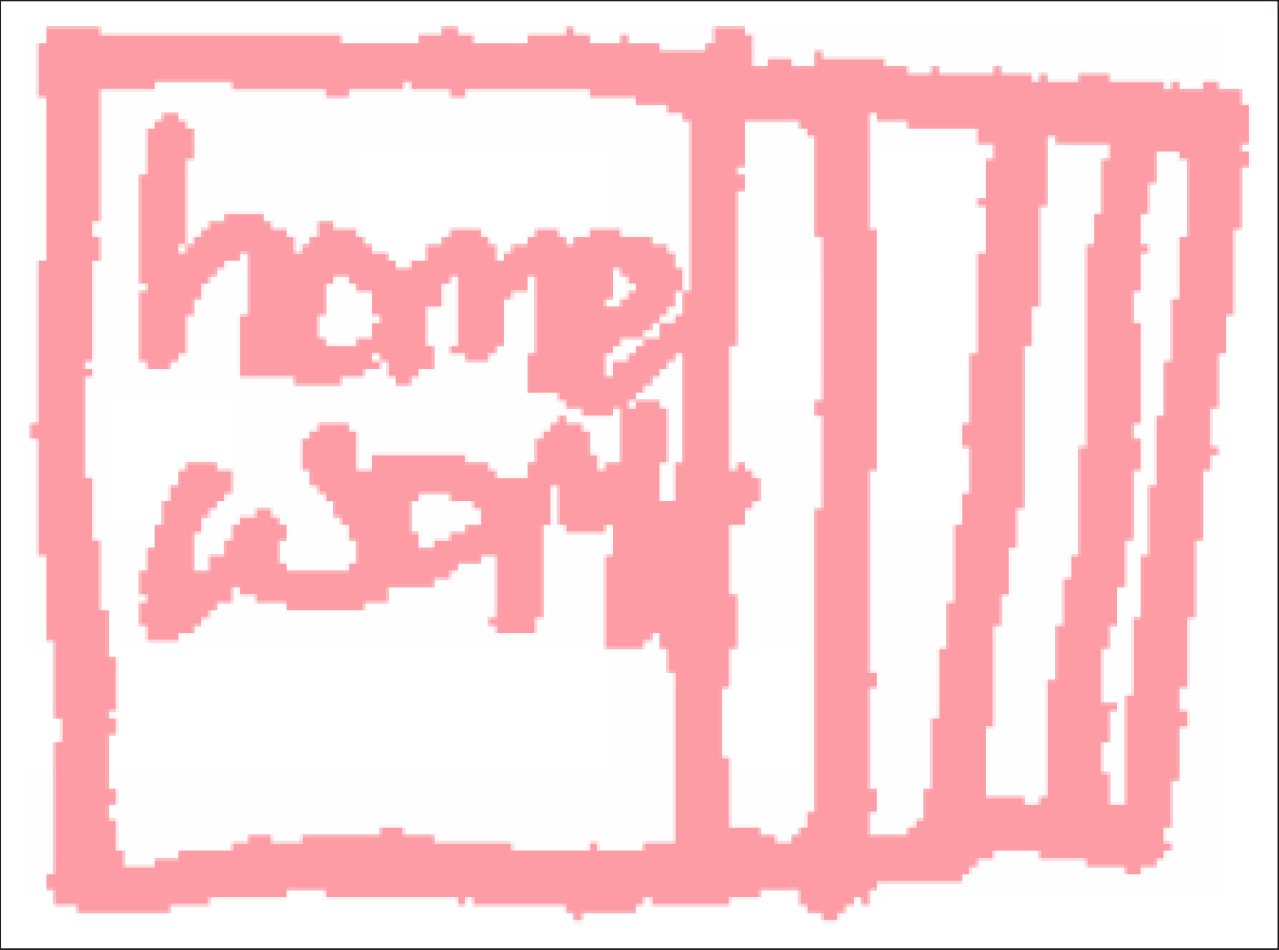
Schoolwork and Homework.

Tessa greatly valued education. She dealt with a challenging upbringing, which included frequently moving schools and living with guardians outside of her nucleus family. Her challenges helped conceptualize her future goals to ensure that her own children would not have the same experiences. Her ideal future was starting up a boutique, finding a husband, and being successful enough to help people in poverty by providing inexpensive services and volunteering. To get to her ideal future, she thought that she had to get to college, believing that would provide the skills and merits she needed to obtain her goals. She was very driven and had the mindset of being a perfectionist: Perfect grades would get her into college, and thus, her perfect future. Anything less than perfection would deny her desired future. Classwork and homework were the barriers to her future and caused her great stress during the 2020–2021 school year. Figure 2 presents a drawing from Tessa illustrating her conflict with schoolwork and homework during the 2020–2021 school year in all learning environments.

During the 2020–2021 school year, a new grading system had been mandated by the district with the justification that it would help parents better understand their children’s grades. All teachers at the high school had to follow a “70–30” guideline. Specifically, 70% of students’ grades had to be based on practice activities, and 30% on quizzes, tests, and projects. “It was a way to pass more kids, plain and simple, by reducing the challenge of passing. It made the classes easier inherently, so to get around the guideline, my homework became harder and worded in a way that students couldn’t just look it up online especially since we had to teach the same amount of material … Our entire math department had the same idea too,” remarked one teacher when reflecting on the 70–30 guideline.

The teachers resisted the new grading policy by providing more rigorous and voluminous homework to ensure students completed the curriculum while not cheating on their homework. Tessa shared that, even when she was in attendance, she had more challenging homework than ever before. She estimated that the average school night of homework involved multiple hours. This was compounded when she was gone. To meet the demands of homework and classwork, she started skipping school to complete the missing work at her own pace. Despite her strategy, her grades started to dip, which brought her great anxiety. Her future was going to be denied. Her racing thoughts worsened the quality of her work and reduced her focus when she was in class, which in turn, continued to damage her grades. Seminar, the school’s designated study hall, was the only time students could receive help during online school. However, one of Tessa’s peers commented on the culture of seminar sharing, “Everyone used seminar as a time to just get away for a bit. The teachers weren’t available even if you needed them.” Even though seminar was presented as a constructive time to get academic help, in practice, nobody, including teachers, followed through with it. Therefore, Tessa had minimal outlets from which to receive help.

The return to in-person learning during hybrid brought a new wave of constraints in relation to grades. Tessa shared her experiences coming to hybrid with mixed emotions:

I was really excited when learning that we would go hybrid, but it kind of faded away because it became like weird and like not really functioning that well, especially for classes like math. And like, I would struggle so much in that class because I was in one day and then home the next day. And [they] changed the lessons to try and like fix everything. Sometimes, we did something in our off days, and then we didn’t. It was really confusing and hard. So, hybrid was really messy. So, once we went to hybrid, of course, like I said, it was really difficult because of all the changes. But I’m still thankful that we can be here in this school because it’s just easier to focus and to learn and see friends.

Hybrid learning also brought a new shift in challenges for teachers, especially with the expectation for the asynchronous day. One teacher remarked, “Before joining hybrid, we were shown some videos of teachers from a neighboring school who were in hybrid, and their comments were that they didn’t really expect kids to work on their asynchronous days. It became our school’s expectation too. So, I guess we were just being told to only teach kids half as much.”

Despite the familiarity of in-person learning during hybrid learning, the pressure to get through the curriculum and seeing what worked/didn’t work resulted in additional stressors for Tessa due to the fluid structures presented. Another student shared about the constraints of hybrid learning commenting that, “I hated hybrid the most because classes were so dense that you could never ask questions. It was one thing to the next thing. We didn’t do anything on our off days, and you had to wait three more school days to ask your teacher questions in class which often meant that you had to wait a week. It was a mess.”

While the amount of homework was the same, there were even fewer opportunities during hybrid for Tessa to receive help because she saw her teachers only once every four days. Returning to full-time in-person learning also failed to mitigate the issue. She rode the bus to school, which meant that she could not receive morning or afternoon help. Therefore, seminar was the only outlet to get help once again during both hybrid and full-time in-person learning. However, due to COVID-19 contact tracing, students were not allowed to travel to other teachers’ seminar during in-person learning to receive help. Students still had to get onto teachers’ live Zoom link to receive help just like during remote learning.

Tessa shared that it was embarrassing to receive help, so students rarely used the tool; the prevailing belief remained that seminar was a time for rest. However, Tessa attempted to receive help for her classes during hybrid only to never see the teachers themselves, thus establishing the belief that the teachers were not interested in helping the students during seminar. One teacher commented that, “Nobody got onto Zoom [during seminar.] So, I always logged off a few minutes into seminar because nobody was using it.”

### Marble

#### Physical Environment (Remote Learning Worsening the Anxiety-ADHD Feedback Loop)

Marble suffers from ADHD and anxiety. She knew that her ADHD made it challenging for her to focus during class time. Therefore, she utilized strategies (e.g., positive self-talk, standing up, getting a drink of water) to help her maintain focus during class. During remote learning, her teachers typically filled in class time by using teacher-led instruction and lectures, which was a common pedagogical theme from the school’s online learning period. An administrator shared his experiences observing teachers during remote learning:

During remote, I zoomed in with teachers. The students’ cameras were off, and there was little interacting face-to-face with teachers, so lots of lecturing happened. The interactions to see facial and body expressions is very critical for teachers to figure out students.

However, efforts were made to attempt student-led instruction, but a series of failures shifted the teachers’ pedagogy to teacher-led instruction. One teacher shared her perspective on attempting student-led instruction before shifting to teacher-led instruction:

We didn’t have the option of asynchronous learning. We had to provide synchronous instruction. It didn’t take long for students to put on blank screens during remote learning. I would never see students. I tried doing hands-on learning, putting them in breakout rooms, discussion prompts, doing polls, journaling outdoors, real-world projects and research, bring an example of ‘this item.’ What would you do if only one student showed me their item they found around their house on Zoom? I was not wanting to be a ‘Here, you are going to watch me lecture’ type of teacher, but I had no choice with the level of their engagement. So, let’s cut the middleman … We had so much stuff to get through.

**Figure 3:**
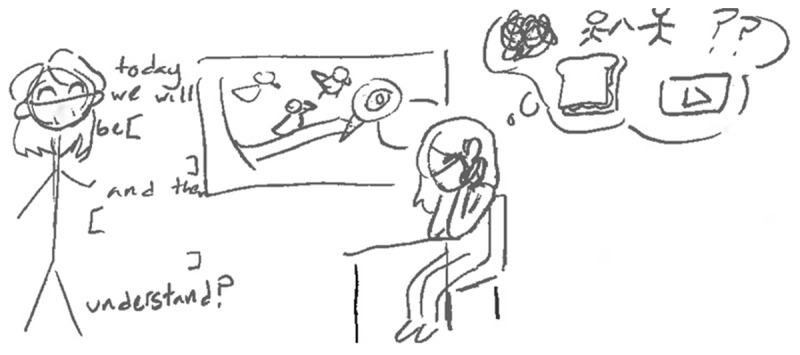
Marble’s Anxiety-ADHD Loop.

The teacher felt that her efforts to bring about student-led instruction strategies during remote learning did not result in productive student learning, so despite knowing the restraints of teacher-led instruction, she started presenting lectures as opposed to open-ended student-led activities. Another student reinforced the teachers’ struggle with student-led and teacher-led instruction, “Nobody would want to interact [with their teachers or peers.] It was a struggle with the teachers obviously. So, then [the teachers] felt like they needed to present.” Marble classified herself as a good student but had challenges focusing during remote learning due to the amount of lecturing. Figure 3 shared her drawing to illustrate her challenges.

Marble expanded on her drawing:

I wrote around here when I drew my thought bubble. It was more just a bunch of random things that I think about every day because … My mind … Every time the teacher will say something, either they say something that reminds me of something, or I just start thinking about something more interesting on accident … It’s just really easy to get distracted by everything, and that includes things outside. So, sometimes, I’ll get distracted by my own thoughts, and sometimes I get distracted because I see things. And then I start thinking about those things a bit too much. And then, my teacher [is] talking with blanks in it because I always managed to either miss the most important information or come back at a time where I missed the information that led up to it. I generally feel more at peace when I’m allowed to do my own things because, even when I am listening to my music and I’m doing my own things, my mind does wander quite a bit. But it’s hard to explain it. But it feels nice – like it feels more natural, and it feels just good to let my mind wander.

The act of missing classwork was detrimental to Marble’s academic performance. She would not understand the material. She would attempt to learn it on her own, but it often resulted in failure. It damaged her self-worth. School was an environment where she was not successful. Marble felt incapable of performing well and felt in conflict. During remote learning, the option of leaving school was easy. All she had to do was leave her conference calls or not show up in the first place. When she did attend remote learning, she struggled focusing on the details of the lecture. Her teachers were not as rigorous as Tessa’s teachers, so she received more time to complete missing work. At other times, she was simply excused from it. The remote learning environment, which predominantly involved lecturing that Marble could not focus on, prevented the opportunities for her to learn. The loss of learning and the downward trend of her grades worsened her anxiety, and her anxiety worsened her focus, which led to poorer grades and more anxiety. Therefore, to escape the anxiety-ADHD feedback loop brought on from remote learning’s lectures, Marble engaged in absenteeism, as it presented a reprieve from her grade-induced anxiety and freedom to divulge into her wondering thoughts.

The switch to in-person learning environments (i.e., hybrid and full-time in-person learning) brought a reduction in teacher-led instruction (i.e., lecturing) and presented more student-led instruction (e.g., projects, labs, hands-on experiences, collaboration). A teacher shared that, “Students were excited to be back in hybrid learning – they were actually talking and interacting with each other. So, I trusted them to do project and collaborative work again.”

Marble felt much better during student-led instruction because she had control over her learning. She also saw the relevance to her learning and understood that the skills would help her, in some capacity, get a job after high school. Also, if her mind wandered, it did not damage her understanding of the content. She felt that the ability to move around and talk with others helped her focus on the task at hand. To that effect, she was also able to learn about content through the in-person experiences, which reduced her feelings of anxiety at school. With lowered feelings of anxiety, Marble successfully attended school more frequently and started looking forward to being in school.

#### Social Capital (School-Based Social Interactions)

Marble’s daily decision to attend school was influenced by social capitals largely due to the social interactions (or lack thereof) within the learning environments during the 2020–2021 school year. The lure of social interactions was a main contributing factor to her daily attendance. She expanded:

I think probably the biggest motivator was seeing friends who you wouldn’t have gotten to see most of the summer because of things going on with them. But yeah, I think the biggest motivator when they were in school was coming to see friends, which is probably why online school was so hard because you wouldn’t be able to see any of your friends that way.

Remote learning did not provide the physical space that allowed her to be around her friends or acquaintances. Even if she had friends in class, they typically had their web cameras off and did not communicate with her. She rarely encountered friendly banter or discussion and felt completely isolated from everyone. A teacher shared the lack of communication between students during remote learning:

I tried to do daily attendance questions that were fun and engaging to start dialogue between students. But I never heard anything. It made my efforts feel very fruitless. They didn’t seem interested in talking to each other.

Marble was unable to make friends or feel connected with anybody, including teachers, due to the lack of social engagement. Her grades worsened, and due to the lack of social connections, she did not feel comfortable asking for help. She felt trapped. When returning to the in-person (i.e., full-time in-person/hybrid) learning environment, she was able to see and be around friends that she had not seen since the start of the pandemic. As a result, she felt more included and connected; school brought her joy which led to an intrinsic desire to attend.

Juxtaposed with her social interactions were academic expectations in relation to school performance. Another teacher remarked on his frustration over the lower performance of some of his students during remote learning:

Starting in October [2020], I had kids who wouldn’t even turn in things that we were working on in class. Things we simply gave them points for attempting and submitting to Schoology. Why were so many kids giving up? I partially blamed the decision on the fact that the students were told they didn’t have to keep their screens on. How could I make sure they were even there if I couldn’t see them? And I know many of them weren’t there. They would log on, and that was the last thing they would do. Nothing we did in class was turned in. No questions were asked or answered by those students – many of which were asked directly. And at the end of the class, they were still on after everyone else had left. They just simply weren’t there. I wasn’t going to give them a passing grade for sleeping! I was giving them about a fourth of what I would have in a normal year. They were just lazy and didn’t want to work. Tough. Their grade would suffer. And suffer they did.

Like Tessa, Marble was typically a strong academic performer in both elementary and middle school. However, her experience with remote learning shifted her beliefs away from being a strong student. Her social circle, including parents, peers, and teachers, saw a decline in her performance. During remote learning, she rarely heard any positive comments about her academic performance from anybody. She felt like her teachers did not treat her the same as before because she had lower grades. She would get called out for a question or with a private message but felt anxious, which led to the impression that her teachers did not like her. She commented that she would, “just walk away [because] I felt like they didn’t like me.” She had received glowing reviews from her previous teachers but felt that her poorer grades led to less affection from her current teachers. She observed the lowering of expectations and then shifted her behavior to meet the lowered expectations. This further reinforced her disengagement with school.

However, the return to in-person learning brought a change in social dynamics, and thus, an improvement in grades. One teacher reflected on the return to in-person learning, sharing: “Starting in hybrid, kids interacted; it felt more normal. [Full-time] in-person felt like normal school. They appreciated school more, and students that did nothing during remote were doing things now. It was a pleasant surprise. I got to know [these students] more too and liked them.” An administrator added, “It was harder for teachers to connect with [students] because [the students] didn’t have to interact with teachers unless they were in sports. It got a lot better as we went from remote to hybrid and [full-time] in-person.” Marble’s grades also improved, which led to a wave of praise and a renewal of confidence; the change in teacher expectations motivated her to attend school more often compared to her remote learning experience.

Thus, the remote learning environment, despite efforts by teachers, failed to provide Marble with meaningful social interactions. Additionally, teachers presented Marble with a deficit mindset due to her lower grades. However, the return of in-person learning led to more social engagement and interactions, more teacher praise, and the promotion of higher academic expectations from her teachers.

## Discussion

The results of this study showed the factors that were influential in shifting the participants’ decision-making toward absenteeism (or school attendance) along with an interpretation of how the factors shifted participants’ daily attendance decision. When reviewing the findings through the ecological agency theoretical framework, the factors situated within the iterative (i.e., past history), practical-evaluative (i.e., environmental capitals and resources), and projective (i.e., aspirations) dimensions motivated participants’ daily attendance decision.

Tessa had endured bullying throughout her academic career, which her schools failed to change. School hurt her; therefore, she disliked the school environment. However, she knew that receiving an education was the pathway to a better future.

Marble liked school before the start of the pandemic. The remote learning environment reversed this trend and made school an unlikable place for her. Her projective dimension was less clear than Tessa’s because her goals were more focused on feeling happy in the near future. Actions that focused on improving her mood were her most desirable outlet even though she understood that schooling was important for her future.

These findings leave the practical-evaluative dimension, which focuses on how the school shifted the participants’ decision-making toward absenteeism (or school attendance). This dimension conceptualizes and clarifies the institutional problems influencing student absenteeism. The school environment failed to protect Tessa from being bullied. The counselors illustrated a shared belief that most reported bullying instances were not bullying. Thus, this approach failed to reduce instances of bullying and reinforced Tessa’s distrust of the school environment. Additionally, the school environments (i.e., remote, hybrid, or full-time in-person) did not provide any viable outlets for Tessa to escape to during her more anxious states beyond her counselor, who was unable to meet with her consistently. All learning environments also failed to provide meaningful academic supports to improve her grades or reduce her grade-induced anxiety despite additional stressors presented from rigorous and voluminous homework (which itself was a collective teacher response to policies related to grades and curriculum).

Remote learning introduced a shift in instruction, which ended in predominantly teacher-led instruction. Marble missed content, became deflated, and gave up. The return to hybrid and full-time in-person learning led to a shift back to student-led instructional practices, an improvement in grades for Marble, and reinforced confidence. The failure of socialization opportunities and minimized academic expectations motivated her absenteeism. She also felt that her teachers had lower expectations for her during remote learning which, when compared to the rest of her schooling experiences, was a completely new and contrary experience for her. It reduced her ambition to perform well at school. However, the in-person learning environment reversed these trends and led to improved attendance.

Our findings support and reinforce previous student absenteeism research, including anxiety leading to absenteeism (e.g., Heyne et al., 2011, 2019; Kearney & Bensaheb, 2006; Melvin & Tonge, 2012), the importance of positive social interactions and social involvement with school attendance (e.g., Keppens & Spruyt, 2017; Kipp & Clark, 2021; Sobba, 2018; Wang & Degol, 2015), the link between bullying and absenteeism (e.g., Grinshteyn & Tang, 2017; Havik et al., 2015), and the need for academic supports in improving attendance (e.g., Thornton et al., 2013).

The present study contributes new knowledge to the student absenteeism literature by revealing how teacher-led instruction restricts access to new learning, which motivated absenteeism and led to lower student efficacy. It also illustrates how lower teacher expectations, especially when the student previously experienced higher expectations, and damaging collective teacher beliefs (i.e., demanding homework as a teacher response to curricular and grading expectations) motivate absenteeism. Further, the findings magnify the need for in-school outlets for students to escape their anxiety. Finally, the present study introduces the manifestation of negative school beliefs due to a lack of bully prevention.

While previous research has illustrated the positive attributes of remote learning (i.e., improved focus compared to in-person learning spaces, better teacher-pupil relationships, more student engagement, and more positive teacher interactions) (Bubb & Jones, 2020), the present study contradicts these attributes (i.e., absentee students in their remote learning context had challenges with concentration, poor teacher-pupil relationships, less student engagement, and fewer positive teacher interactions).

### Implications

The implications of the above findings and discussion point to the need to improve the problematic institutional/school dynamics that influence absentee behaviors. In turn, the improved structures may better afford student attendance. The participants’ experiences amplified the problem of anxiety and illustrate the need for both socioemotional interventions to improve their emotional state and a space for students to briefly escape the classroom environment and manage their emotions before returning to their classes. Counselors or trusted adults within the school can be established as sources for students to manage their anxiety. These outlets provide an avenue for students to manage their feelings of anxiety. It also illustrates the need for proactive and sustained bully prevention measures. Some schoolwide interventions include improving one-on-one relationships, implementing and maintaining prosocial education, adding schoolwide support and supervision, and promoting sustained ownership among all stakeholders (Cohen et al., 2009; Cohen & Freiberg, 2013; Durham & Connolly, 2017).

The role of the teacher is an important measure in preventing bullying (De Luca et al., 2019). However, the perceptions of the staff in the current study imply that they were unaware of bullying during remote learning because they did not see students interacting or failed to recognize bullying by attributing actions of bullying as retaliation for previous actions (i.e., staff’s deficit mindset toward the bullied student). Bullies use school personnel’s nonintervention to normalize the bullying behavior (Campaert et al., 2017), which Tessa alluded to (i.e., “And so, it got to the point that I really thought it was normal.”). Raising teacher awareness and competencies through opportunities to practice interventions, observing successful interventions, and opportunities to learn and share bullying interventions can help prepare teachers to be proactive in preventing bullying (Campaert et al., 2017). An asset perspective (i.e., changing the environment) as opposed to a deficit perspective (i.e., bullied students’ fault led to the bullying) is necessary for staff to identify and resolve instances of bullying.

The grading policy and curricular expectations during the 2020–2021 school year led to new teacher actions (i.e., teacher-led instruction, demanding homework), specifically through the teachers’ resistance to the new grading policy and adherence to the curricular expectations. This infers the need for teacher voice in curricular/policy decision-making with new policies and expectations to promote teacher agency that conforms to rather than resists new institutional policies (Bridwell-Mitchell, 2015).

Providing academic supports can reduce grade-induced anxieties through improved reliability (i.e., follow through) of tutoring services, specific study halls for certain disciplines, peer-peer tutoring opportunities, external paid tutors, and flexibility within curriculum pacing to meet students’ immediate needs.

The dynamics within the classroom also influenced the participants’ daily attendance decisions in the full-time in-person, hybrid, and remote learning environments. The use of student-led instruction at the start of remote learning was transformed into teacher-led instruction within the remote learning environment and led to gaps of content, which harmed participants’ self-worth, efficacy, and understanding of the material. The teachers reported using student-led instruction through digital tools and the home learning environment, which has been found to enhance learning and engagement in a remote setting (e.g., Bubb & Jones, 2020). However, the teachers observed that student-led instruction led to low student engagement. Collaboration is an indispensable feature of student-led instruction (Iversen et al., 2015), but the teachers did not observe any collaborative efforts from students. To that end, teachers found lecturing to be more productive in meeting curricular needs. The teachers shifted back to student-led instruction starting in hybrid because they saw improvement in student engagement and success. One teacher shared that, “We know how to do [in-person student-led instruction] well. We could comment immediately on what a student does [during hybrid and full-time in-person learning], and they got better grades than remote. I put back in labs, discussions, projects that I cut during remote.”

The use of student-led instruction (e.g., laboratories, discussions, projects, cooperative learning) returned during hybrid and full-time in-person learning, which led to high student engagement and success, as Marble demonstrated. Therefore, lecturing led both participants to engage in absenteeism during remote learning, which implies that other pedagogy beyond lecturing is needed to promote attendance and not absenteeism. Moreover, the teachers’ challenges with student-led instruction illustrate a lack of efficacy with their online student-led instruction, a need to instruct teachers on quality online student-led practices, as well as the need for further research into improving teacher efficacy for remote learning (which has been reported to be problematic) (Cardullo et al., 2021). The study also presents a need for research into effective pedagogical strategies to improve active engagement in the remote setting for both non-absentee and absentee students.

Additionally, lower teacher expectations, especially when compared to previous experiences of higher teacher expectations, motivated student absenteeism. For example, Marble felt that her teachers did not like her and did not expect her to do well. When Marble showed her teachers her capabilities, the teachers’ behaviors and actions changed and increased her confidence in relation to academic rigor. In turn, she felt empowered to perform which was reinforced with praise when she did perform well. This illustrates the importance of high teacher expectations and should be the norm for teachers to help promote school attendance. Once again, the lack of teacher-student engagement during remote learning was remedied during in-person learning settings and emphasizes the need for strategies to improve teacher-student rapport during remote learning.

Finally, positive peer-peer socialization opportunities within school and classroom environments are paramount to promoting school attendance regardless of the learning environment. Providing peer collaboration time within the classroom and schoolwide extracurricular programs may provide a space for natural socialization to occur between students especially in remote learning environments, which did not have any constructive avenues for peer socialization time beyond sports. It may also promote the synthesis of friendships and the opportunity to build meaningful teacher-peer rapport. During hybrid and full-time in-person learning, I observed that clubs started to come back such as Ping Pong, Geology, and Chess Clubs; remote learning environments also need to have extracurricular programs and synchronous in-class socialization to provide opportunities for students to socialize with their peers.

## Limitations, Recommendation for Future Research, and Conclusion

The study was limited by the specific context, the unique experiences of the participants, and the contextual factors presented at the school. Moreover, while case studies are useful for generalizing bounded cases, they are unable to extend their findings to larger, unbounded populations (Creswell & Poth, 2018).

Future research should continue to explore different or specific groups of absentee students (e.g., backgrounds, levels of academic achievement, experiences) as well as different school locales, contexts, and levels. Finally, future studies should further explore teacher efficacy and effective pedagogy in the remote learning environment.

School absenteeism is problematic and worsened due to the COVID-19 pandemic. The present study employed an ecological agency approach to student absenteeism to capture the decision-making of absentee students in a suburban U.S. school during the COVID-19 pandemic, which included remote, hybrid, and full-time in-person learning contexts. The findings illustrate that the lack of social opportunities during remote learning, bullying and the nonintervention of bullying, the lack of spaces to escape anxiety, the absence of academic supports, lecturing during remote learning, and lower teacher expectations promoted student absenteeism. Therefore, more socialization opportunities in all learning environments, a consistent commitment to bullying interventions and belief that students are being bullied, prescribed spaces to reduce grade-induced anxiety, academic supports, more research into effective student-led remote learning pedagogy/teacher efficacy in remote learning environments, teacher voice in policy development and implementation, and an asset mindset for staff members can help improve the school and classroom dynamics to promote attendance and reduce absenteeism as we continue to navigate the unique challenges presented by the COVID-19 pandemic.
